# Medawar and Hamilton on the selective forces in the evolution of ageing

**DOI:** 10.1007/s40656-021-00476-6

**Published:** 2021-11-25

**Authors:** Stefano Giaimo

**Affiliations:** Department of Evolutionary Theory, Max Planck Institute for Evolutionary Biology, August-Thienemann-Straße 2, Plön, 24306 Germany

**Keywords:** Age, Reproductive value, Senescence, Theory

## Abstract

Both Medawar and Hamilton contributed key ideas to the modern evolutionary theory of ageing. In particular, they both suggested that, in populations with overlapping generations, the force with which selection acts on traits declines with the age at which traits are expressed. This decline would eventually cause ageing to evolve. However, the biological literature diverges on the relationship between Medawar’s analysis of the force of selection and Hamilton’s. Some authors appear to believe that Hamilton perfected Medawar’s insightful, yet ultimately erroneous analysis of this force, while others see Hamilton’s analysis as a coherent development of, or the obvious complement to Medawar’s. Here, the relationship between the two analyses is revisited. Two things are argued for. First, most of Medawar’s alleged errors that Hamilton would had rectified seem not to be there. The origin of these perceived errors appears to be in a misinterpretation of Medawar’s writings. Second, the mathematics of Medawar and that of Hamilton show a significant overlap. However, different meanings are attached to the same mathematical expression. Medawar put forth an expression for the selective force on age-specific fitness. Hamilton proposed a full spectrum of selective forces each operating on age-specific fitness components, i.e. mortality and fertility. One of Hamilton’s expressions, possibly his most important, is of the same form as Medawar’s expression. But Hamilton’s selective forces on age-specific fitness components do not add up to yield Medawar’s selective force on age-specific fitness. It is concluded that Hamilton’s analysis should be considered neither as a correction to Medawar’s analysis nor as its obvious complement.

## Introduction

We are all familiar with the generalized deterioration that members of our species and domesticated animals experience late in life. To this deterioration, we refer to as ‘ageing’ or ‘senescence’ in everyday language and it is different from the mere passing of time (Nathan, 2021). Ageing is particularly conspicuous in the human life history, where it involves both mental and physical aspects (Boniolo, 2021; Green & Hillersdal, 2021) and influences one’s moral perspective and evaluations (Jecker, 2021). While biomedicine is active in understanding how to defer ageing (Blasimme, 2021), philosophy attempts to conceptualize ageing (Green & Hillersdal, 2021; Lemoine, 2020; Sholl, 2021) and to understand the moral limits to set on our ability to mould the lifespan, e.g. Wareham (2021).

The first investigations into the causes of ageing date back at least to Aristotle (Finch, 2010). Evolutionary biology has also long been interested in ageing. Darwin published the first edition of his work on the origin of species in 1859. Twenty-two years later, August Weismann (1891) gave a lecture on the duration of life at a meeting of the Association of German Naturalists. This lecture is traditionally considered as the first attempt to explain at length why ageing evolves (Rose, 1991). However, the published edition of Weismann’s lecture contains a small earlier note on the subject by Alfred Russel Wallace. Roughly, Weismann proposed that ageing is the result of cellular mechanisms that put an upper limit to the regenerative ability of somatic tissues by inhibiting indefinite cell duplication. In his view, natural selection would favour these mechanisms to accelerate population renewal. According to Weismann, no individual can perfectly repair damage of environmental origin. For example, a wound in a tissue leaves a scar, which is not as functional as uninjured parts of the same tissue. As living longer implies a longer exposure to hazards, unrepaired damage should be found in large amount in long lived individuals. Weismann then deemed “necessary that new and perfect individuals should continuously arise and take their place,” as “[w]orn-out individuals are not only valueless to the species, but they are even harmful, for they take the place of those which are sound.” (Weismann, 1891, p. 24). In his theory, selection led death and ageing to evolve to shorten lifespan “by the amount which was useless to the species” (Weismann, 1891, p. 25). Weismann’s theory was later criticized and dismissed (Comfort, 1956; Williams, 1957), especially for assuming group selection (Williams, 1957), although in subsequent writings Weismann no longer invoked that assumption (Kirkwood & Cremer, 1982). Garson (2021) offers a philosophical account on how some early tenets of Weismann’s view nevertheless appear to reverberate in the ideas of later scholars.

A modern evolutionary theory of ageing was then proposed exclusively assuming individual level selection (Hamilton, 1966; Kirkwood & Rose, 1991; Medawar, 1958; Williams, 1957). A cornerstone of this theory is that selection acts with different force on traits of adult individuals depending on the age at which these traits are expressed: the later the age of expression, the weaker the selection on the trait. This theory is still key to our understanding of how ageing evolves and has inspired much empirical research. This research has confirmed some predictions and, at the same time, highlighted limitations of the theory. Overcoming these limitations or revisiting the validity of some predictions is a central task in current theoretical work on the evolution of ageing. Flatt and Partridge (2018) give a recent overview on these topics. More generally, evolutionary biology keeps having a lively research interest in ageing.

The present paper looks at a specific methodological and interpretative issue in the history of the modern evolutionary theory of ageing. The aim is to reconsider the relationship between the analysis of the force of selection by Peter B. Medawar and that by William D. Hamilton. Although the work of the latter is often seen as an obvious continuation of the former, a belief is commonly expressed in the literature that Hamilton perfected Medawar’s insightful, yet ultimately erroneous analysis of why the selection force declines in populations with overlapping generations. I will argue that this belief is wrong: both analyses agree in some of the ways in which they quantify the weakening of the selection force with age. However, I will also suggest there are reasons not regard one analysis as being the obvious complement of the other either.

Here is a brief scheme of what follows. Initially, the background is set by introducing how ageing is defined in evolutionary biology. Concomitantly, I present the standard way, which is generally credited to Hamilton (1966), in which the decline of the force of selection with age is quantified. Then, I illustrate the common belief about Hamilton’s analysis being a correction of Medawar’s. Subsequently, I reconstruct Medawar’s thinking on the force of selection in the evolution of ageing. On the basis of this reconstruction, I revisit the belief according to which Medawar’s analysis of the selection force is erroneous when compared to Hamilton’s. Finally, I highlight the actual differences between the two analyses.

## Selective forces and the evolution of ageing

### Ageing

**Fig. 1:**
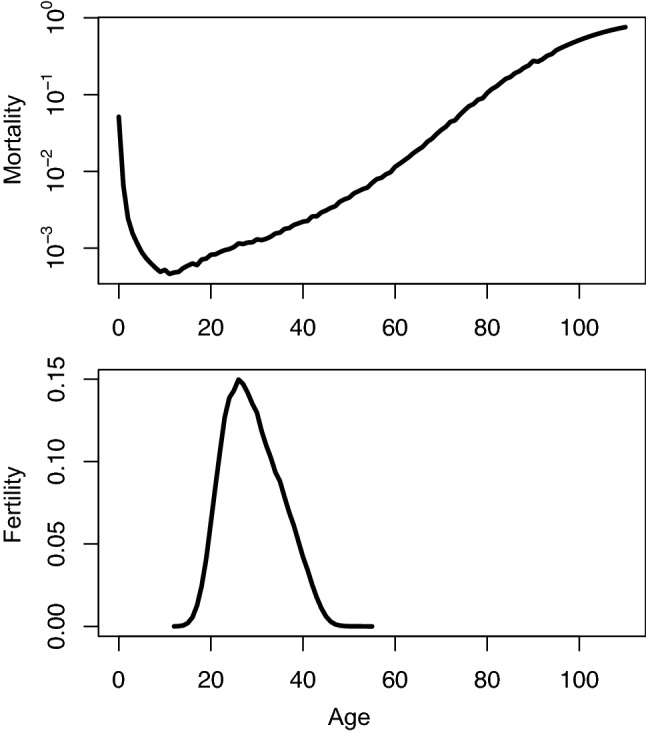
Mortality and fertility in women, Italy 1954. Mortality data are from the Human Mortality Database (https://www.mortality.org/). Mortality at each age is here approximated by the central death rate. Fertility data are from the Human Fertility Database (https://www.humanfertility.org/). Both databases were accessed on the 19th Dec 2019

Ageing or, equivalently, senescence is defined in evolutionary biology as a progressive degeneration of bodily faculties that starts at some point during adulthood and causes survival and fertility to decline with age (Charlesworth, 1980; Comfort, 1956; Medawar, 1958; Rose, 1991). Plotting human female mortality and fertility against age (Fig. 1) exemplifies this concept. Following adolescence, human mortality increases with age, while fertility, after reaching a maximum before age 30, wanes and, eventually, hits zero as menopause begins. Ageing is observed in numerous species, but some appear not to senescence (Jones et al., 2014). In some species including humans, the fly and the worm, the acceleration of mortality with age seems come to a halt at very late ages (Barbi et al., 2018; Vaupel et al., 1998), although some concerns have been raised about the reliability of data on supercentenarians in inferring any real deceleration in mortality, e.g. Newman (2018). However, proponents of what I refer to as the modern theory regarded ageing as a virtually universal phenomenon with anecdotic exceptions (Comfort, 1956; Hamilton, 1966; Medawar, 1958).

### The declining force of selection

The key idea in the modern theory is that ageing evolves because the force with which selection acts upon traits expressed at some age declines the later the age under consideration (Hamilton, 1966; Medawar, 1958; Williams, 1957). Here is a rough explanation of where this decline originates from. Evolutionary biology takes survival and reproduction as the two main activities of the living: individual survival is evolutionarily meaningless without reproduction, which is the way in which individuals guarantee the survival of their genes through generations. Fitness is a combination of survival and fertility throughout the lifespan. But an individual’s future survival and reproduction do not count towards fitness as much as present survival and reproduction. In fact, survival to some age is always less likely than survival to any earlier age because of the possibility of intervening death. Moreover, an offspring later in life is a smaller relative contribution to the population than an earlier offspring if the population has meanwhile increased in size. Therefore, the later in life a gene exerts its effect the less likely this effect will actually matter to realized fitness. As a consequence, detrimental mutations with late effect may be only scarcely opposed by selection, while mutations with early beneficial effect and later detrimental consequences may increment net fitness (Medawar, 1958; Williams, 1957). The progressive incorporation of mutations of either type in the population would eventually provoke a deterioration of late life bodily faculties, i.e. ageing.

As is well known, the idea that selection would weaken with age is anterior to what are generally regarded as the first at-length discussions of it by Medawar (1958) and Williams (1957). Both these authors mention Haldane, who for example remarked that “[i]n man there is good evidence that arteriosclerosis and other senile diseases are largely genetically determined. It is natural that such genes should accumulate as the result of mutation, for there is no selection against genes which act after the reproductive period” (Haldane, 1941, p. 113). In exposing his thoughts on the force of selection at different ages, Medawar (1958, p. 44) even states that he has “had the most valuable advice from Professor J B.S. Haldane, some of whose ideas are presented here as if they were my own.” But Medawar also acknowledges the influence of Penrose (Medawar, 1958, p. 67), who noted that “when a disease has early onset it may disable the individual and effectively diminish his fertility. This tends to exclude affected individuals, in whom onset is early, from the class of parents in any pedigree material” (Penrose, 1947, p. 126). Similarly, Williams (1957, p. 399) sees his ideas on senescence as reminiscent of those of Bidder (1932), among others. To Bidder it is generally attributed the theory that ageing results from a developmentally programmed arrest of individual growth (Kirkwood & Cremer, 1982; Reznick et al., 2002). Should this growth continue, it would guarantee–according to Bidder–persistent somatic renewal and perpetual, or even increasing, fertility. But he also noted that “[i]f primitive man at 18 begat a son, the species had no more need of him by 37, when his son could hunt food for the grandchildren. Therefore the dwindling of cartilage, muscle, and nerve cell, which we call senescence, did not affect the survival of the species; the checking of growth had secured that by ensuring a perfect physique between 20 and 40. Effects of continued negative growth after 37 were of indifference to the race” (Bidder, 1932, p. 585).

### Quantifying the force of selection

However, the foregoing ideas were not cast in formal terms. Hamilton (1966) is usually credited with having given the first mathematically explicit formulation of the idea that selection declines with age (Rose, 1991; Rose et al., 2007; Williams & Taylor, 1987). The rough explanation of this idea I gave above is an attempt at verbalizing his theory. Hamilton derived a number of expressions meant to capture different selective forces acting upon genetic variation whose phenotypic manifestations are linked to individual age. These expressions would collectively show that selection tends to attenuate through the lifespan of individuals. Here, his main results are reviewed with reference to a haploid population to avoid the complications introduced by mating system and diplody. Moreover, the population is assumed not to be subject to density dependence, i.e. there is no limit to how much the population can grow. These assumptions reflect Hamilton’s 1966 treatment.

Some notation is needed. We let *l*(*a*) be the fraction of a cohort of newborns that is still alive at age $a\ge 0$. The quantity *l*(*a*) declines monotonically with age *a*, as no new individual is added to the cohort and death progressively erodes the initial cohort. We normalize *l*(*a*) so that $l(0)=1$, i.e. the initial cohort size is set to unity, as its actual size does not matter so long as this is very large. Mortality $\mu (a)=-l'(a)/l(a)$ at age *a* for this cohort is the relative rate at which the cohort shrinks with age because of individual deaths. It gives the absolute value of the slope of the function *l*(*a*) at each age point *a* relative to the function value at that point. Let *b*(*a*) be the fertility of individuals aged *a* and let $\beta$ be the first reproductive age, i.e. fertility at any age prior to $\beta$ is zero, while it is positive for ages greater than $\beta$ (the existence of a post-reproductive period will not be considered here). Suppose age-specific mortality and fertility in the population are constant: they may change with individual age but not with time so that distinct individuals of the same age exhibit the same vital rates although they may be alive at different time points. Mathematical demography (Keyfitz & Caswell, 2005) shows that, eventually, as a result of constant age-specific mortality and fertility the population approaches a stable age distribution: the relative fraction of same aged individuals in the population approaches a constant value with time, the population relative growth rate $N'(t)/N(t)$, where *N*(*t*) is population size at time *t*, also approaches a constant value *r*. At demographic stability, the logarithm of population size is a linear function, with slope *r*, of time and Euler-Lotka equation holds:

1 $$\begin{aligned} \int _{x=\beta }^{x=\infty }l(x)b(x)e^{-rx}\text {d}x=1. \end{aligned}$$

The importance of this equation is in making stable population growth *r* an implicit function of age-specific mortality and fertility. Applying the tools of differential calculus to Eq. (1), one can examine how *r* responds to small changes in mortality and fertility at some age, i.e. the sensitivity of *r* to such changes. This is exactly what Hamilton (1966) did. Following Fisher (1930) in equating *r* with fitness, Hamilton considered a whole “spectrum of forces” (Hamilton, 1996, p. 90) by studying how fitness would react to mutations of different kind. He differentiated *r* in Eq. (1) in a number of ways to obtain: the sensitivity $s_{\mu }(a)$ of *r* to a small additive effect on mortality in an infinitesimally small interval around a single age *a*; the sensitivity $s_{\mu \bullet }(a)$ of *r* to a small additive effect on mortality from age *a* onwards; and the sensitivity $s_{b}(a)$ of *r* to a small additive effect on fertility at a single age *a*. (He also considered mutations with effect on mortality from one age to another, but we will not consider this result here.) (Hamilton, 1966, Eqs. 9, 11 and 25) obtained the following expressions for these sensitivities,

2a $$\begin{aligned} s_{\mu }(a)= -\frac{1}{T}\int _{x=a}^{x=\infty }l(x)b(x)e^{-rx}\text {d}x, \end{aligned}$$

2b $$\begin{aligned} s_{\mu \bullet }(a)= \int _{x=a}^{x=\infty }s_{\mu }(x)\text {d}x, \end{aligned}$$

2c $$\begin{aligned} s_{b}(a)=\frac{l(a)e^{-ra}}{T}, \end{aligned}$$

where *T* is the generation time. Equation (2a) shows that the effect of a change in mortality at some age *a* is proportional to residual fitness at that age, as captured by the integral of survivorship and fertility over remaining ages discounted by population growth. When a change in mortality is imposed from age *a* onwards, Eq. (2b) shows that the resulting fitness effect is equivalent to adding up the fitness effects of age-specific changes in mortality for all relevant ages. The expression in Eq. (2c) shows that the effect of a change in fertility at age *a* relates to the stable fraction aged *a*, which is proportional to the number of those who have survived from birth up to age *a*, i.e. *l*(*a*), relative to how much the population has grown since their birth, i.e. $e^{ra}$.

These sensitivities illuminate evolutionary considerations in age-structured populations. Suppose a new mutation emerges in the population. Mutants have slightly different survival or fertility at some age. Mutations that increase either of this quantity will make mutant growth superior to resident growth, while mutations that decrease them will reduce mutant growth when compared to the resident. Keeping the magnitude of the mutational effect constant regardless of the age at which this is exerted, the ultimate probability of establishment of these mutations can be shown to depend on the magnitude of the above sensitivities (Charlesworth, 1973, 1975). The age pattern of these sensitivities then yields the force of selection at different ages, as they tell us how the evolutionary fate of mutations with effect linked to age depends on this age.

Compare two mutations with effect on mortality at two distinct reproductive ages *y* and $z>y$. The corresponding fitness sensitivities are obtained by setting *a* equal to *y* and to *z* in Eq. (2a) for an age-specific effect and in Eq. (2b) for a continuing effect with onset age *a*, respectively, and, then, taking absolute values. In both cases, fitness always is more sensitive to the mutation with effect at age *y* due to integration over a larger positive region. If the two mutations affect fertility at different ages, when the population is not going extinct (i.e. $r\ge 0$), the mutation with earlier effect has an impact at least as strong on *r* as the other mutation, as in Eq. (2c) the fraction *l*(*y*) of a newborn cohort surviving to age *y* is at least as large as the fraction *l*(*z*) surviving to any later age *z*.

But Hamilton also worked out the mathematics behind other selective forces. Consider new mutations that act to modify the age *a* at which genes that are already present in the population at some appreciable frequency (i.e. those connected with heritable genetic disorders and mutations that are already gone to fixation) exert their effect. The expressions

3a $$\begin{aligned} g_{\mu }(a)= s_{\mu }'(a)=T^{-1}l(a)b(a)e^{-ra}, \end{aligned}$$

3b $$\begin{aligned} g_{\mu \bullet }(a)= s_{\mu \bullet }'(a)=-s_{\mu }(a), \end{aligned}$$

capture the selection force to change the age of action of a gene with effect on mortality only around age *a* and the selection force to change the age *a* of onset of a gene with lasting effect on mortality, respectively (Hamilton, 1966, Eqs. 15 and 16). Both expressions are obtained using the fundamental theorem of calculus on Eqs. (2a)–(2b). However, note that in Hamilton’s 1966 paper, these expressions lack the factor *T* of generation time. While Hamilton (1966, p. 21) was sceptic that genes of the former kind would actually exist, he considered plausible the existence of modifiers of the age of onset of traits. Since $g_{\mu \bullet }(a)=-s_{\mu }(a)$ and $-s_{\mu }(a)$ is a decreasing function of reproductive age $a>\beta$, the selective force on age-of-onset modifiers always declines with reproductive age.

In Hamilton’s view, his Eqs. (2) and (3b) would collectively show the inexorable decline of the force of selection with reproductive age and, by extension, the inevitability of ageing in evolution. On a side note, indicators of the force of selection that are alternative to Eqs. (2a) and (2c) exist. And they fail to support Hamilton’s view that the force of selection always declines with age (Baudisch, 2005). Further research has also highlighted that selective forces acting to change the age at which other genes exert their effect or start doing so (Eq. 3) are unlikely to be evolutionarily important (Charlesworth, 1980). However, these and related topics will not be considered in full here. The focus of the present work is on a specific issue in the history of the modern theory of ageing, and not in the more contemporary ramifications of this theory.

## A common belief

In the literature about the ecology and evolution of senescence, Medawar (1958) always occupies a prominent place whenever the basis of the modern theory of ageing are reviewed. In particular, he is generally credited with the first at length discussion of why the selection force should decline with age and how this decline should be measured. Sometimes the work of Hamilton on ageing is seen as a natural continuation of Medawar’s (Hitchcock & Gardner, 2020; Rose et al., 2007). But in the literature the qualification is also found that, however largely insightful, Medawar’s analysis is ultimately wrong, as it does not coincide with Hamilton’s, which is regarded as the correct analysis, e.g. Caswell and Shyu (2017, p. 57), Charlesworth (1973, p. 309; 2000, p. 928), Crow (2002, p. 1315), Emlen (1970, p. 589), Hamilton (1966, p. 13), León (1976, p. 17), Michod (1979, p. 545), Moorad et al. (2019, p. 520), Monaghan et al. (2008, p. 372), and Rose et al. (2008, p. 365).

However, in comparing Medawar and Hamilton, different authors make this qualification based on different premises and to different effects. Here, four main versions of this qualification are summarized:

A$$_1$$
Medawar’s mistake was to view the selection force on new mutations with effect at age *a* that appear in the population as given by Fisher’s reproductive value *v*(*a*) at age *a*, e.g. Crow (2002), Charlesworth (1973, 2000), Hamilton (1966), Monaghan et al. (2008). This would be the force acting against (in favour) of a new mutation with detrimental (beneficial) effect felt at a specific age or set of ages. Using the notation above, this force would be quantified by
  4 $$\begin{aligned} v(a)=v(0)\dfrac{e^{ra}}{l(a)}\int _{y=a}^{y=\infty }l(y)b(y)e^{-ry}\text {d}y, \end{aligned}$$
  (Fisher, 1927). Here, *v*(*a*) gives the residual reproduction for an individual of age *a* where births *b*(*y*) to this individual when aged $y\ge a$ are discounted both by cumulated population growth, i.e. $e^{r(y-a)}$, over the time interval $y-a$ and by the probability, i.e. *l*(*y*)/*l*(*a*), the individual survives up to age *y* given that has survived to *a*. In the words of Fisher (1930, p. 27), who applied it human populations, *v*(*a*) measures “[t]o what extent will person of this age, on average, contribute to the ancestry of future generations.” Note that *v*(*a*) is expressed relative to the reproductive value *v*(0) of a newborn. By choosing a specific value for *v*(0), reproductive value can be arbitrarily scaled.
A$$_2$$
Medawar’s mistake was to see the selection force on traits with effect at age *a* as reducible to the stable population fraction *c*(*a*) aged *a*, e.g. Caswell and Shyu (2017), with
  5 $$\begin{aligned} c(a)=\frac{l(a)e^{-ra}}{\int _{x=0}^{x=\infty }l(x)e^{-rx}\text {d}x}=bl(a)e^{-ra}, \end{aligned}$$
  where *b*, the multiplicative inverse of the integral in the denominator, is the population birth rate (Keyfitz & Caswell, 2005).
A$$_3$$
Medawar’s mistake was to view the selection force on traits with effect at age *a* as proportional to newborn survival *l*(*a*) up to that age, e.g. Moorad et al. (2019).
A$$_4$$
Medawar’s mistake was to believe that Fisher’s reproductive value *v*(*a*) at age *a* would capture the selection force on modifiers of the age *a* of action, or of onset, of already established genes with effect on mortality, i.e. Emlen (1970), Hamilton (1966), León (1976) and Michod (1979). This would be the selective force that tends to change the age at which deleterious (beneficial) alleles exert their effect to reduce (enhance) their consequences on fitness.

**Fig. 2:**
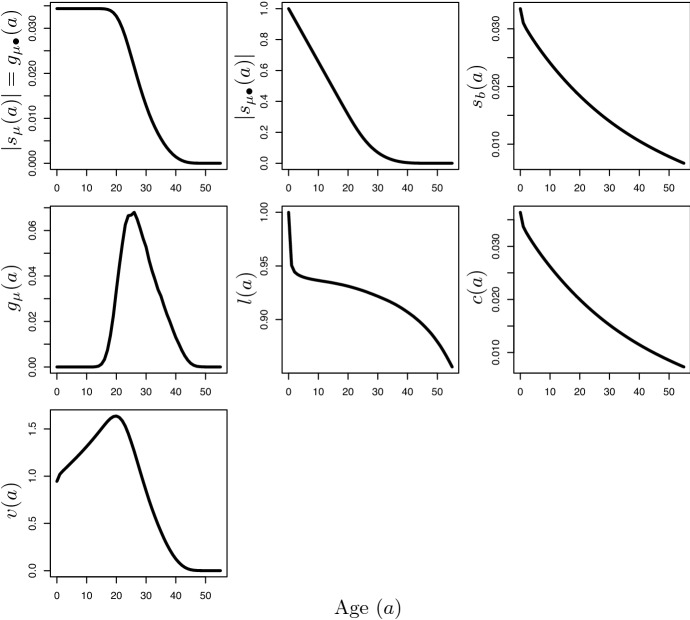
The first four plots (reading row-wise from left to right) report Hamilton’s main forces of selection against age, see Sect. 2.3 and equations therein. The three subsequent plots report newborn survivorship *l*(*a*), stable age distribution *c*(*a*), and reproductive value *v*(*a*) against age with *v*(0) scaled so that $\int c(a)v(a)=1$. All quantities are computed on the same data as Fig. 1. Age (*x*-axis) is shown from birth up to just before menopause

In general, the quantities *v*(*a*), *c*(*a*) and *l*(*a*) do not correspond to Hamilton’s selection forces as expressed by Eqs. (2)–(3). The sole exception is *c*(*a*), which is proportional to $s_{b}(a)$ as a comparison between Eqs. (2c) and (5) reveals. Figure 2 shows how *l*(*a*), *c*(*a*) and *v*(*a*) may behave differently with age compared to Hamilton’s expressions. Hence, putting aside the proportionality between *c*(*a*) and $s_{b}(a)$, if Medawar had claimed a decline in the selection force with age by regarding this force as proportional to any of these quantities, he had certainly been wrong. But the disagreement between those who judge Medawar’s analysis on how selection operates at different ages as ultimately incorrect when compared to Hamilton’s is evidence that it is not an entirely settled issue what the actual upshot of Medawar’s analysis really is.

## Medawar on the selection force

What were Medawar’s ideas about the force of selection at different ages? He addressed the problem of the evolution of ageing in two separate essays: ‘Old Age and Natural Death’ and ‘An Unsolved Problem of Biology’, which were published in 1946 and 1952, respectively. It then seems helpful to read back these essays to answer our question. Both are rich in ideas about ageing. However, we shall limit attention only to those sections that are directly relevant to our question. The two essays are collected along others in Medawar’s book *The Uniqueness of the Individual* (Medawar, 1958). We shall then conveniently refer to their pages as they appear in this book.

### Selective forces in *Old Age and Natural Death*

In ‘Old Age and Natural Death’, Medawar first reviews a number of theories about ageing. Among them, he discusses Weismann’s theory (see the Sect. 1), of which he values the motivating idea that ageing can be explained from an evolutionary perspective. However, Medawar is critical of Weismann’s supposition that selection would remove old individuals from the population by increasing their likelihood to die because they are damaged and not as efficient as young ones in propagating the species. What Medawar finds problematic is that Weismann’s theory assumes that ageing is already present in the population (Medawar, 1958, p. 19). Therefore, Weismann’s theory can at most explain the evolution of a faster rate of ageing and not the evolutionary origin of ageing.

It is to explain how ageing can originate that Medawar asks us to imagine “a population that is potentially immortal” (p. 36), in Medawar’s peculiar sense that senescence is absent and individuals of different ages are indistinguishable under all biologically and demographically relevant aspects. In particular, all individuals in the population experience a nonzero, age-independent mortality throughout their life and their fertility is also age-independent (pp. 36–37). Medawar supposes that the population has reached its stable age distribution and is stationary in size ($r= 0$). At this demographic equilibrium, he notes, the stable fraction aged *y* in his hypothetical population is always larger than the stable fraction aged $z>y$. Because of constant mortality $\mu =\mu (a)$ at all ages, stationary growth ($r= 0$) and the relationship between $\mu (a)$ and *l*(*a*), using Eq. (5) we can in fact see that, in Medawar’s thought experiment,

6 $$\begin{aligned} c(y)=bl(y)e^{-ry}=be^{-y\mu }>be^{-z\mu }=bl(z)e^{-rz}=c(z). \end{aligned}$$

Medawar then specifies that

What is important from our point of view is that the contribution which each age-class makes to the ancestry of future generations decreases with age. Not because its members become progressively less fertile; on the contrary, it is one of our axioms that fertility remains unchanged, so that the reproductive value *per head* is constant; but simply because, as age increases, so the number of heads to be counted in each age-group progressively falls. It is at least as good a guess as Weismann made, that the process of senescence has been genetically moulded to a pattern set by the properties of this ‘immortal’ age distribution. (Medawar, 1958, pp. 37–38)

In his hypothetical population, Medawar says, the reproductive value *v*(*a*) of an individual of age *a* is independent of the individual’s age. This can be checked by noting that, when fertility $b(a)=b$ is constant at all ages *a* and newborn survivorship to age *a* is $l(a)=e^{-a\mu }$, Euler-Lotka equation reduces to $r=b-\mu$. Using this fact, reproductive value (Eq. 4) similarly reduces to $v(a)=v(0)b/(\mu +r)=v(0)$. But, Medawar observes, in the population each age class, i.e. the fraction of same aged individuals, contains a greater reproductive value than any later age class. This is because the class contains a larger number of individuals and individual reproductive value is independent of age. Thus, paraphrasing Fisher’s definition of reproductive value, Medawar concludes that each age class contributes more to the future ancestry of the population than any later age class in his “immortal” population.

He then goes on discussing different sorts of mutations that may spread in the population as a result of their age pattern of action and the age class distribution of reproductive value. For example, he says

It is by no means difficult to imagine a genetic endowment which can favour young animals only at the expense of their elders; or rather, at their own expense when they themselves grow old. A gene or combination of genes that promotes this state of affairs will under certain numerically definable conditions spread through a population simply because the younger animals it favours have, as a group, a relatively large contribution to make to the ancestry of the future population. [...] This does not imply that a late-acting gene which confers selective advantage cannot spread through the population. It can indeed do so; but very much more slowly than a gene which gives evidence of itself earlier on. The later the time in life at which it appears, the slower will be its rate of spread; and the rate in the end becomes vanishingly small. (Medawar, 1958, p. 38)

Both in this quotation and the previous one, Medawar emphasizes the fact that, in assessing the fitness consequences of new mutations with effects that become first apparent at specific ages, he is considering the reproductive value of age classes. In other words, it would seem that he takes *c*(*a*)*v*(*a*) as the quantity that captures the selection force at age *a*, where the relative number *c*(*a*) of individuals aged *a* at demographic stability is multiplied by the individual reproductive value *v*(*a*) at that age. It should be noted that Gardner (2019) apparently was the first to observe that Medawar equated the age-specific selection force with age-class reproductive value. Medawar then appears to combine the fact that, in his population model, individual reproductive value is identical at any two ages *y* and $z>y$, i.e. $v(y)=v(z)$, with the fact that the population fraction aged *y* and *z* differ (Eq. 6) to conclude that

7 $$\begin{aligned} c(y)>c(z)\Rightarrow c(y)v(y)>c(z)v(z), \end{aligned}$$

which would indicate that the force of selection declines with age even when ageing is not present in the population. This fact, according to him, should explain how senescence may evolve in the first place. However, we should also note that Medawar does not clearly separate effects on mortality from effects on fertility. He generically speaks about mutations conferring a selective advantage and looks for the force of selection on overall fitness at each age. But he is not explicit as to whether mutational effects should be limited to a single age or, once apparent, lasting for longer.

Having explained how ageing may evolutionarily originate, Medawar then looks at how selection operates when ageing is already present in some form. To this effect, he drops a number of assumptions he had made to build his hypothetical “immortal” population. In particular, he supposes that individual fertility is no longer age independent, it instead declines at late ages and so must, too, the individual reproductive value (p. 38), as this gives the relative reproductive prospects left to an individual of a given age. Furthermore, individuals are not immediately fertile, they first have to mature up to some age in which reproduction becomes possible (p. 39). Medawar then imagines that, in this population, there are already established genes that produce their effect at some point in the life of the individual or that have an effect of varying intensity through the lifespan. The following are his thoughts on the selective forces operating on these genes:

It may be shown that if the time of action or rate of expression of such genes is itself genetically modifiable, then, if the gene confers selective advantage, its time of action or of optimal expression will be brought forward towards youth, as it spreads through the population. If, by contrast, the gene is ‘disadvantageous’, then its time of action or threshold of unfavourable expression will be pushed onwards in life while it is being eliminated from the population. [...] Neither process can come into operation unless the fertility of the population declines with age, so that the reproductive value of its members falls; [...] Because of the hazards to which baby animals are exposed [...] the reproductive value of the individuals always rises to a maximum before eventually it falls; and it is at the epoch of this maximum, therefore, that the ‘precession’ of favourable gene effects will automatically come to halt. It is not surprising, then, to find that in human beings the ‘force of mortality’ is lowest just when the reproductive value would in the members of a primitive society be highest—in the neighbourhood of the fourteenth or fifteenth years of life. (Medawar, 1958, p. 39)

According to Medawar, the selective force acting to change the age at which already established genes, both detrimental and beneficial, exert their effect is strictly related to the individual reproductive value *v*(*a*) at different ages *a*. In non-decreasing populations ($r\ge 0$) where individual fertility drops at late ages, as in humans, reproductive value first increases with juvenile age at least up to the first reproductive age. Only at some later point it declines towards zero as fertility also goes to zero. The initial increase in reproductive value can be seen from Eq. (4) by noting that, at juvenile ages ($a<\beta$), *v*(*a*) reduces to $v(0)e^{ra}/l(a)$ in virtue of Euler-Lotka equation and the newborn fraction *l*(*a*) surviving to age *a* monotonically decreases with increasing *a*. A numerical illustration is in Fig. 2.

In Medawar’s view, the timing of deleterious genes would be shifted away from the peak of reproductive value in either direction, i.e. towards birth or towards late life, while this peak would attract the timing of beneficial genes. This seems to be his interpretation of Fisher’s idea, referenced to in a footnote (p. 39), that “[i]t is probably not without significance [...] that the death rate in Man takes a course generally inverse to the curve of reproductive value ” (Fisher, 1930, p. 29).

Therefore, Medawar appears to consider that at least two separate selective forces exist that contribute to the evolution of ageing. The first is the force that acts in favour or against the incorporation of newly arisen genetic variants depending on the age at which their fitness effects become apparent. Its magnitude over age would be captured by the age class reproductive value. This force, in his view, can explain how ageing can originate where it was previously absent. The second force is the one that operates when ageing is already present to some extent so that the individual reproductive value already exhibits an increase to some intermediate peak to then progressively drop to zero, at least for human-like life histories. The magnitude of this second force at different ages is captured by the individual reproductive value at those ages. Pushed by this force, traits that are already found in the population would tend to be expressed close to the peak of individual reproductive value, when they are beneficial, and far from it, chiefly late in life, when they are detrimental.

### Selective forces in *An Unsolved Problem of Biology*

The essay ‘An Unsolved Problem of Biology’ is, according to Medawar (1958, p. 16), “a lengthy footnote” to the passages of ‘Old Age and Natural death’ that we have quoted before. In this later essay, Medawar asks us again to consider a hypothetical “population of potentially immortal individuals” (p. 58), where immortality is to be understood, once more, in terms of age-independent mortality and fertility (p. 60). The population is assumed stationary. This time, however, Medawar envisages individuals in the population as test-tubes, and not as animals, in the hope to convey the idea that they remain unchanged with age. Test-tubes may spontaneously break (death) and an experimenter replaces them (birth). As in his previous essay, Medawar notes that the fraction of individuals aged *y* is larger than the fraction aged $z>y$ (p. 60), see Eq. (6) and explanations therein, and that

[a]lthough each individual test-tube takes an equal share of the ancestry of the future population, each age-group most certainly does not. The older the age-group, the smaller is its overall reproductive value. (Medawar, 1958, pp. 60–61)

He then elaborates on “some of the consequences of this decline in the reproductive value of older age-groups” (p. 61) and states that

There is a constant feeble pressure to introduce new variants of hereditary factors into a natural population, for ‘mutation’, as it is called, is a recurrent process. Very often such factors lower the fertility or viability of the organisms in which they make their effects apparent; but it is arguable that if only they make them apparent late enough, the force of selection will be too attenuated to oppose their establishment and spread. (Medawar, 1958, pp. 61–63)

Here, Medawar’s view is reiterated that the selection force on mutations with fitness effect at some age declines with their age of effect because the age class reproductive value declines with age. The individual reproductive value instead is assumed constant with age. As in the previous essay, Medawar does not clearly differentiate between mutations with effect on mortality and those with effect on fertility. He considers effects on overall fitness at a given age.

Medawar subsequently examines how selection tends to change the age at which genes that are already present in the population have their effect. As an example, Medawar considers Huntington’s chorea, a heritable degenerative disorder of humans that manifests itself usually after the third decade of life and is generally lethal within 20 years from onset. Thinking about this and similar genetic disorders, Medawar comments that

If hereditary factors achieve their overt expression at some intermediate age of life; if the age of overt expression is variable; and if these variations are themselves inheritable; then natural selection will so act as to enforce the postponement of the age of the expression of those factors that are unfavourable, and, correspondingly, to expedite the effects of those that are favourable—a recession and a precession, respectively, of the variable age-effects of genes. [...] The theorem in the form in which I have just put it does not depend upon the existence of a post-reproductive period; it only requires that the reproductive value of each age-group should diminish with increasing age. I have argued that this must necessarily diminish even with a population of potentially immortal and indeterminately fertile individuals, provided only that they are subject to real dangers of mortality. In such a population a younger age-group must necessarily outnumber an older, for the older represents the residue of those who have been longer exposed to mortal hazards. (Medawar, 1958, pp. 67–68)

It would seem that, here, Medawar sees the selection force to modify the age at which genes have their effect as related to the reproductive value of that age class. This would be a discrepancy with his belief expressed in ‘Old Age and Natural Death’ that this force should be expressed by the individual reproductive value. However, a few lines later, he appears to go back to his prior idea by stating that “the precession of the time of action of genes comes to a standstill at the epoch when the reproductive value is at a maximum, and it is then that senescence should be expected to begin” (Medawar, 1958, p. 69).

In summary, in ’An Unsolved Problem of Biology’, Medawar reaffirms the same views he held in *Old Age and Natural Death* except for introducing the ambiguity on whether individual reproductive value or age-class reproductive value would capture the selective force optimizing the age of expression of genes already present in the population. On a side note, Medawar seems to regard a form of external mortality to be a necessary condition for the force of selection to decline. Wensink et al. (2017) provide a clear discussion of the role of this form of mortality in the evolution of ageing.

## Assessing the belief

We are now in a better position to revisit the belief that Hamilton’s analysis of selective forces in populations with overlapping generations corrects Medawar’s insightful, yet ultimately wrong analysis. Let us look back at A$_{1}$–A$_{4}$ in Sect. 3 and assess their merits.

A$_{1}$ is incorrect. The problem with it is that Medawar consistently regards age class reproductive value *c*(*a*)*v*(*a*), and not individual reproductive value *v*(*a*), as the quantity that would capture the selection force on new mutations appearing in the population with effect at age *a*. Medawar intentionally analyses population models so constructed that individual reproductive value is constant with age. Yet the age class reproductive value declines with age. This decline, in Medawar’s view, corresponds to the decline in the selection force on newly arisen mutations the later their age of expression.

A$_{2}$ is incorrect to the extent that it equates the stable age distribution *c*(*a*) with the quantity that Medawar regards as indicative of the selection force on new mutations appearing in the population with effect at age *a*. He equates this force with age class reproductive value, which combines stable age distribution and individual reproductive value. A$_{2}$, however, correctly points to an important aspect to Medawar’s analysis. In the case of a population where individual reproductive value is age independent, Medawar attributes the decline of this force with age with the diminishing fraction of same aged individuals found in the population the later the age under consideration. In Medawar’s scheme, this is the relevant case to analyse in order to understand the evolutionary origin of ageing. Therefore, the problem with A$_{2}$ is in not separating the way in which Medawar proposes to quantify the selection force at different ages (i.e. with age class reproductive value) from the reason behind the age-related decline of this quantity in “immortal” populations (i.e. the shape of the stable age distribution).

A$_{3}$ is incorrect for the same reasons as A$_{2}$. In fact, newborn survivorship *l*(*a*) is proportional to the stable age distribution in stationary populations ($r=0$) such as those considered by Medawar, see Eq. (5). Similarly to A$_{2}$, A$_{3}$ also rightly points to a relevant aspect to Medawar’s analysis: it is the decline of *l*(*a*) with age under a nonzero mortality regime that explains why in “immortal” populations of stationary size the fraction *c*(*a*) of *a*-aged individuals also diminishes with increasing *a*.

A$_{4}$ is correct as long as we look at ‘Old Age and Natural Death’. There, Medawar indeed views the force of selection that moves around the age at which genes act as directly influenced by the trajectory of reproductive value over age. In Hamilton’s analysis this force is instead shown proportional to either $g_{\mu }(a)$ or $g_{\mu \bullet }(a)$ (Eq. 3), depending on whether genetic effects (assumed to be on mortality) are either limited to a single age or lasting, respectively. Both these expressions differ from individual reproductive value (see, e.g., Fig. 2). However, A$_{4}$ misses the inherent ambiguity of Medawar in ‘An Unsolved Problem of Biology’, where this selective force is also quantified with the age class reproductive value.

## Medawar and Hamilton

Having discussed A$_{1}$-A$_{4}$, it remains to understand what the true relationship between Medawar and Hamilton is when it comes to quantify the selective forces behind the evolution of senescence. In this respect, we should recall that it was Hamilton himself who initiated the belief that his analysis would supersede that erroneous of Medawar. As Hamilton (1996, p. 88) recounted it, his interest in how selection operates at different ages was prompted by his reading *The Genetical Theory of Natural Selection* by Fisher (1930). In particular, he was struck by Fisher’s suggested relationship between mortality and individual reproductive value (see above and below). Hamilton (1996, p. 88) considered his 1966 paper as an “attempt to find out what those comments by Fisher could mean.” He held in high regard the result of such attempt: “a unique case where I thought I had seen farther, or at least more clearly, on one issue than the giant on whose shoulders I had for so long tried to balance” (Hamilton, 1996, p. 89). Hamilton believed that Medawar had made the same error as Fisher in equating the selection force on mortality with individual reproductive value:

Medawar in his 1952 lecture combined the development of a model which did lead him to the outlines of what we believe to be the correct theory with tentative adherence to a logically inconsistent opinion about the forces operating in the immature period. This latter seems to have been taken over uncritically from Fisher (1930, p. 29) who had written that he thought it “probably not without significance [...] that the death rate in man takes a course generally inverse to the curve of reproductive value”. As may be seen from the diagrams given in this paper a human curve of reproductive value [...] rises to a maximum shortly after the attainment of reproductive maturity, while the curve of force of mortality [...] has a minimum at or slightly before it. [...] We hope to make it clear that the correspondence to which Fisher draws attention in the above statement is really largely trivial and that in the context to which they were restricting themselves the idea which he tacitly and Medawar explicitly assumed is without foundation. (Hamilton, 1966, p. 13)

To expose what he perceived as “the absurdity of the idea that reproductive value outlines the forces of selection tending to prevent senescence” (Hamilton, 1996, p. 23), he devoted two entire sections of his 1966 paper to detail how his expressions $s_{\mu }(a)$ and $g_{\mu \bullet }(a)$, i.e. Eqs. (2a) and (3b) in the present work, which capture the selective forces on new mutations with age-specific effect on mortality and on age-of-onset modifiers, respectively, do not correspond to individual reproductive value *v*(*a*). In particular, he built a model of a population of organisms that experience constant mortality and exponentially increasing fertility from the age of first reproduction (Hamilton, 1966, pp. 23–25). In this model, *v*(*a*) continually increases with reproductive age and, yet, ${|{s_{\mu }(a)}|}=g_{\mu \bullet }(a)$ decreases.

It would then seem that Hamilton attached special importance to $s_{\mu }(a)$ and $g_{\mu \bullet }(a)$ among his indicators of the age-specific selection force to show that this must decrease with age. Notably, most later literature has concurred with him in deeming $s_{\mu }(a)$ in Eq. (2a) as perhaps his most notable finding in relation to the evolution of senescence, e.g. Baudisch (2005), Caswell (2007), Charlesworth (1993), Flatt and Schmidt (2009), Flatt and Partridge (2018), Kirkwood and Holliday (1979), Partridge and Barton (1993), and Rose et al. (2007), although some consider $s_{\mu \bullet }(a)$ in Eq. (2b) to be more relevant, e.g. Abrams (1991).

Somewhat ironically, however, precisely the two results Hamilton held in highest regard are susceptible of being expressed in terms of age class reproductive value. Using Eqs. (2a), (3b), (4), and (5), one has that

8 $$\begin{aligned} \begin{aligned} {|{s_{\mu }(a)}|}&=g_{\mu \bullet }(a)\\&=\frac{1}{T}\int _{x=a}^{x=\infty }l(x)b(x)e^{-rx}\text {d}x\\&=bl(a)e^{-ra}\frac{e^{ra}}{l(a)bT}\int _{x=a}^{x=\infty }l(x)b(x)e^{-rx}\text {d}x\\&=c(a)v(a) \end{aligned} \end{aligned}$$

see also Caswell, (2010, p. 534), Hitchcock and Gardner (2020, SI p. 6) and Lande (1982, Eq. 13b) where, for the scaling of reproductive value in Eq. (4), *v*(0) is set equal to $(bT)^{-1}$. As already stated, Medawar regarded the age class reproductive value as the quantity that would indicate the force of selection at different ages on new mutations affecting fitness at that ages. Notably, developments of evolutionary modeling for age-structured populations subsequent to Hamilton have vindicated, although without directly acknowledging, Medawar’s intuition that the selection force on age-specific fitness is proportional to age class reproductive value (Engen et al., 2011, 2012; Taylor, 1990; Taylor & Frank, 1996). In one instance, Medawar also appears to regard such quantity as directly relevant to how selection operates on age-of-onset modifiers. The reformulation in Eq. (8) then shows that what Hamilton took as two crucial indicators of the selection force at different ages and the one that Medawar suggested only differ by a proportionality factor. In this respect, note that Medawar (1958, p. 61) appears to adopt the convention that $v(0)=1$. Therefore, contrary to the common belief that Hamilton perfected the insightful, yet erroneous analysis of this force by Medawar, some of their key ideas overlap. In particular, parts of the mathematics they used to capture the decline in selection with age are formally equivalent.

Undoubtedly, Hamilton’s analysis is mathematically superior in terms of transparency and generality, i.e. it applies to stable populations with any mortality and fertility schedule. Medawar, instead, proposed a mostly verbal analysis that is more limited in scope (i.e. stationary “immortal” populations) and requires some interpretative effort to be fully understood. This may explain the divergences in opinion among later scholars about Medawar’s contribution to our understanding of why the selection force should decline with age. Intriguingly, Hamilton (1966) played with the relationship between *v*(*a*), *c*(*a*) and ${|{s_{\mu }(a)}|}=g_{\mu \bullet }(a)$. In a passage (p. 22), he referred to the quantity

9 $$\begin{aligned} w(a)=\int _{x=a}^{x=\infty }l(x)b(x)e^{-rx}\text {d}x, \end{aligned}$$

which is the numerator in ${|{s_{\mu }(a)}|}$ and $g_{\mu \bullet }(a)$, as the expected number of offspring after age *a* when offspring are weighted by population growth. But he added that “*w*(*a*) can be considered to measure more exactly what Williams (1957) meant by ‘reproductive probability.’ Unfortunately, it seems impossible to have a phrase which combines this brevity with greater precision, but ‘expected reproduction beyond age *a*‘ is at least more explicit.” Columns of Table 1 of Hamilton (1966) report both *w*(*a*) and the stable age distribution *c*(*a*) normalized so that $c(0)=1$ for all fertile ages of Taiwan women in 1906. The last column of this Table gives the individual reproductive value *v*(*a*) at each age, which is therein stated to be obtainable directly by diving the column of *w*(*a*) with the column of *c*(*a*). About these data and the reproductive values of women and men in the same population that are plotted in his Fig. 3, he also commented (p. 31) that the assumed normalization of individual reproductive value is such that

10 $$\begin{aligned} \int _{x=0}^{x=\infty }v(x)c(x)\text {d}x=1, \end{aligned}$$

where *c*(*a*) is normalized so that its integral over age equals 1. This is precisely the normalization required in Eq. (8). Yet, for some reason, Hamilton failed to explicitly identify ${|{s_{\mu }(a)}|}$ or $g_{\mu \bullet }(a)$ with age class reproductive value, the quantity Medawar had proposed to quantify the selection force on new mutations with effect linked to age.

But how far does the agreement between Medawar and Hamilton actually go? It seems prudent to state that the agreement is only partial. Putting aside modifiers of age-specific genes, Medawar proposed to quantify the force of selection on age-specific fitness with the age class reproductive value. He did not clearly distinguish how selection would operate on mortality and fertility separately at each age. Hamilton made such distinction, but he did not attempt to quantify the selection force on overall age-specific fitness. As it turns out, the expression for the selection force on age-specific mortality coincides with the expression for the selection force on age-specific fitness. But this formal equivalence should not obscure the different biological meanings of these two expressions. Medawar and Hamilton were looking for a quantification of different, however related, selective forces.

In this respect, Hitchcock and Gardner (2020, SI p. 6) seem to implicitly suggest that Hamilton’s analysis might exactly complement Medawar’s. Accordingly, Hamilton would also have taken the age-class reproductive value as quantifying the selection force at each age and his indicators of selection on age-specific mortality and fertility would represent the two components of this force. Using a discrete-time model (Hitchcock & Gardner, 2020, SI Eqs. 14-6), the decomposition of the selection force at age *a* takes the following form:

11 $$\begin{aligned} c_{a}v_{a}=\dfrac{\sum _{i=a}l_{i}b_{i}e^{-ri}}{T}=\dfrac{l_{a}b_{a}e^{-ra}}{T}+\dfrac{\sum _{i=a+1}l_{i}b_{i}e^{-ri}}{T}, \end{aligned}$$

where we have adapted our notation from Eq. (8) by using subscripts for age to distinguish the quantities in this expression from the corresponding ones for continuous time. In Eq. (11), the reproductive value $c_{a}v_{a}$ of age class *a* is decomposed in the sensitivity of *r* to a proportional change in the number $b_{a}$ of offspring produced per individual of age *a*, i.e. the first term on the right-most hand side, and the sensitivity of *r* to a proportional change in the probability of surviving from age *a* to age $a+1$, i.e. the second term on the right-most hand side. These two terms are the time-discrete counterparts of $b(a)s_{b}(a)$ and ${|{s_{\mu }(a)}|}$, see Eqs. (2a) and (2c), respectively. (Note that, in the discrete-time model, unlike in continuous time, the selection force against mortality between ages *a* and $a+1$ takes the same form as the selection force on fitness at age *a*, but the two forces differ by a term.)

The decomposition in Eq. (11), which in different guises is implicit in other studies of age-specific selection (Engen et al., 2011; Lande, 1982), is undoubtedly correct. However, it seems historically inaccurate to attribute this decomposition to the work of Medawar and Hamilton for two main reasons. First, it is hard to find an attempt by Hamilton (1966) to quantify the force of selection on age-specific fitness. As stated before, some of his indicators of more specific selective forces (against age-specific mortality and on age-of-onset modifiers) happen to coincide with the force of selection on age-specific fitness. But, again, formal equivalence does not imply equivalence of meaning. Second, while $s_{\mu }(a)$ is Hamilton’s indicator of the force of selection on mortality, Hamilton did not consider $b(a)s_{b}(a)$ as an indicator of the force of selection on age-specific fertility. Hitchcock and Gardner (2020) recognize the absence of $b(a)s_{b}(a)$ in Hamilton (1966). However, they seem to consider this quantity somehow coherent with his work. Similarly, Lande (1982, p. 610) does not clearly distinguish $b(a)s_{b}(a)$ from $s_{b}(a)$ while referring to the work of Hamilton (1966). In this regard, we should recall that the quantity $b(a)s_{b}(a)$ only appears in Hamilton (1966) because it is equivalent to $g_{\mu }(a)$ in Eq. (3b), which captures the selection force on modifiers of the age of action of an established gene with effect on mortality only around age *a*. As already mentioned before, Hamilton (1966) quickly dismissed the occurrence of such modifiers as implausible (p. 21) and did not consider this selective force any longer. Hamilton only took $s_{b}(a)$ as the selection force on age-specific fertility, his Eq. (25). He certainly deemed this result as “preliminary” (p. 42) because he believed not “so plausible that a gene could simply add an element of fertility at a given age without affecting the rest of the schedule as it is that a gene might cause the elimination of a single element of mortality.” For this reason, he sketched out (p. 43) the potential effects of genes altering the entire shape of the fertility distribution over age under some constraint, e.g. constant mean. It remains unclear why Hamilton did not examine $b(a)s_{b}(a)$, which captures the effect of a proportional change in age-specific fertility as a potential selective force. After all, his indicator $s_{\mu }(a)$ corresponds to the force acting on a proportional effect on survival from one age interval to the next. As noted by Baudisch (2005), what is unfortunate about his neglect of $b(a)s_{b}(a)$ is that this quantity does not in general decline with age, as the panel for the equivalent quantity $g_{\mu }(a)$ in Fig. 2 shows, where even an increase with age is possible. What is relevant for our purposes is that this selective force does not seem coherent with the overall thesis of Hamilton (1966, p. 12) that his results would be “[a] basis for the theory that senescence is an inevitable outcome of evolution” because “even under [...] utopian conditions selection is still so orientated that, given genetical variation, phenomena of senescence will tend to creep in” (p. 25). Thus, only $s_{b}(a)$ should be regarded as Hamilton’s indicator of the force of selection on fertility. Consequently, the indicators of the selection force on age-specific fitness components that Hamilton proposed do not add up to yield the selection force on age-specific fitness, which instead was the quantity of interest for Medawar. It might then be simplistic to view Hamilton’s results as an obvious complement to Medawar’s analysis.

## Conclusions

Medawar occupies a prominent place in the modern evolutionary theory of ageing. This is mostly due to his elaborated discussion of how the force of selection is supposed to decline with age. But, I have argued, both the details of his view on this force and its relation to Hamilton’s view on the same topic, which has long been considered the orthodoxy in the field, are not universally appreciated. Some authors regard Medawar’s basic ideas as insightful, yet ultimately wrong when compared to Hamilton’s. This is not true. Hamilton’s indicator of the force of selection on mortality, a result that both he and later literature in the field have held in highest regard, is of the same mathematical form as Medawar’s proposed indicator of the selection force on age-specific fitness. Other authors see Hamilton’s analysis as the coherent and natural complement to Medawar’s analysis. But this appears simplistic. Despite formal agreement in some of their mathematics, Medawar and Hamilton attached different meanings to the same mathematical expression. While Medawar tried to express the selective force on age-specific fitness, Hamilton instead proposed a full spectrum of selective forces only operating on fitness components, i.e. mortality and fertility. But Hamilton’s selective forces on fitness components do not add up to yield Medawar’s selective force on age-specific fitness.

## Data Availability

Data are available in the public website indicated in the figure legends.

## Code Availability

Code used to generate figures is available upon request from the authors.
